# Improved Spatial Resolution in Modeling of Nitrogen Oxide Concentrations in the Los Angeles Basin

**DOI:** 10.1021/acs.est.3c06158

**Published:** 2023-11-30

**Authors:** Katelyn A. Yu, Meng Li, Colin Harkins, Jian He, Qindan Zhu, Bert Verreyken, Rebecca H. Schwantes, Ronald C. Cohen, Brian C. McDonald, Robert A. Harley

**Affiliations:** † Department of Civil and Environmental Engineering, University of California, Berkeley, Berkeley, California 94720, United States; ‡ Chemical Sciences Laboratory, NOAA Earth System Research Laboratories, Boulder, Colorado 80305, United States; § Cooperative Institute for Research in Environmental Sciences, University of Colorado, Boulder, Colorado 80309, United States; ∥ Department of Chemistry, University of California, Berkeley, Berkeley, California 94720, United States

**Keywords:** air pollution, emission inventory, motor vehicles, satellite

## Abstract

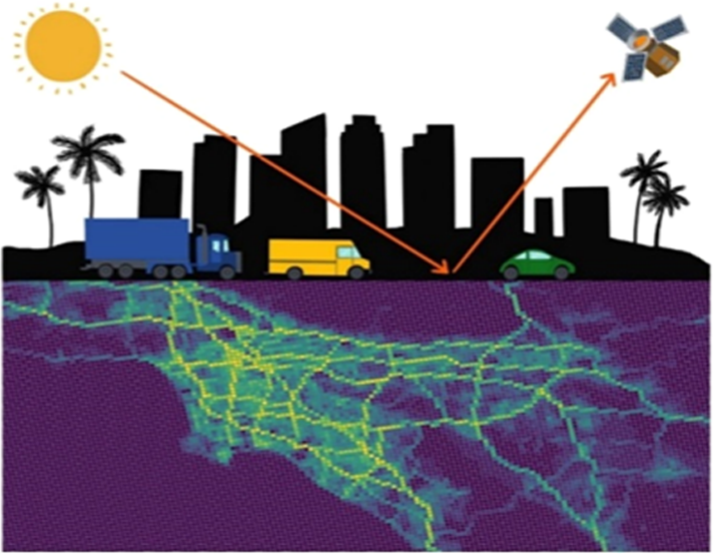

The extent to which emission control technologies and policies have reduced anthropogenic NO_*x*_ emissions from motor vehicles is large but uncertain. We evaluate a fuel-based emission inventory for southern California during the June 2021 period, coinciding with the Re-Evaluating the Chemistry of Air Pollutants in CAlifornia (RECAP-CA) field campaign. A modified version of the Fuel-based Inventory of Vehicle Emissions (FIVE) is presented, incorporating 1.3 km resolution gridding and a new light-/medium-duty diesel vehicle category. NO_*x*_ concentrations and weekday–weekend differences were predicted using the WRF-Chem model and evaluated using satellite and aircraft observations. Model performance was similar on weekdays and weekends, indicating appropriate day-of-week scaling of NO_*x*_ emissions and a reasonable distribution of emissions by sector. Large observed weekend decreases in NO_*x*_ are mainly due to changes in on-road vehicle emissions. The inventory presented in this study suggests that on-road vehicles were responsible for 55–72% of the NO_*x*_ emissions in the South Coast Air Basin, compared to the corresponding fraction (43%) in the planning inventory from the South Coast Air Quality Management District. This fuel-based inventory suggests on-road NO_*x*_ emissions that are 1.5 ± 0.4, 2.8 ± 0.6, and 1.3 ± 0.7 times the reference EMFAC model estimates for on-road gasoline, light- and medium-duty diesel, and heavy-duty diesel, respectively.

## Abstract 2

A high-resolution fuel-based inventory is used as an input in a coupled weather-chemistry model and evaluated against aircraft and satellite. On-road emissions are found to be the largest source sector of nitrogen oxides in the South Coast Air Basin.

## Introduction

Nitrogen oxides (NO_*x*_ = NO + NO_2_) are highly reactive air pollutants that are produced primarily from the combustion of fossil fuels. Exposure to ground-level NO_2_ is associated with increased mortality due to respiratory and cardiovascular diseases, and NO_*x*_ is a major precursor to tropospheric ozone (O_3_), which has negative impacts on human health and the environment.^1−4^ Nitrogen oxides also react in the atmosphere to create acid rain and particulate matter in the form of aerosol nitrates.^4^ To manage the human health and environmental impacts of NO_*x*_, an improved understanding of NO_*x*_ emission sources and the distribution of NO_*x*_ in high-population areas is needed.

In the United States, the development of emissions control technologies has been driven by laws and regulations aimed at reducing air pollution, with major changes starting in 1963 with the Clean Air Act and its subsequent amendments. The primary technologies to reduce NO_*x*_ emissions from vehicles involve devices that convert NO_*x*_ emissions into water and N_2_. The widespread adoption of catalytic converters has significantly decreased the NO_*x*_ emissions from gasoline vehicles since the 1990s. As a result, heavy-duty diesel trucks became the largest mobile source of NO_*x*_ emissions as light-duty gasoline emissions declined and diesel fuel sales continued to grow;^5−7^ these trends were reinforced due to ineffective diesel NO_*x*_ emission controls in the 1990s.^8^ Selective catalytic reduction (SCR) systems were introduced on new heavy-duty diesel engines starting in 2010. SCR systems use a urea solution to convert NO_*x*_ to N_2_ and have been shown to reduce in-use NO_*x*_ emissions from heavy-duty diesel engines by >75%.^9−14^ However, the effectiveness of these systems in reducing NO_*x*_ emissions under real-world driving conditions can be impaired at times due to factors such as extended idling and low exhaust temperature leading to inactivation of the emission control system.^11,14^

The extent to which adoption of more effective NO_*x*_ control technology has affected emission trends in the United States remains unclear. Field campaigns, satellite-based inventories, and chemical transport models have produced results that highlight uncertainties in NO_*x*_ emission inventories, including how much NO_*x*_ is being emitted and the extent to which NO_*x*_ emissions have decreased in recent years. Estimates of NO_*x*_ based on the MOtor Vehicle Emission Simulator (MOVES) emission model overestimated NO_*x*_ when compared to field campaigns in the Baltimore-Washington area and in the southeast US.^15−17^ The MOVES model also estimated larger decreases in NO_*x*_ emissions than NO_2_ satellite retrievals suggest.^18^ These differences are coupled with the fact that while precursor emissions to ozone have decreased over the past several decades, ozone concentrations in urban areas have declined relatively slowly and background ozone in the northern hemisphere has been slowly increasing.^19,20^

Many urban areas in North America are transitioning toward NO_*x*_-limited ozone formation regimes, implying that NO_*x*_ emission reductions are increasingly necessary to reduce ozone.^21,22^ The Los Angeles area is one of the largest and most populated urban areas in the United States, with air pollution problems that are linked to local topography, high volumes of vehicle traffic, and two major ports that handle nearly 30% of all imports and exports over the water in the United States.^23^ Ozone concentrations in the region remain high despite major reductions in ozone precursors, and as a result, the South Coast Air Basin (SOCAB) was designated “extreme nonattainment” for ozone in 2018.^7,24^

Chemical transport models (CTMs) have been useful tools for understanding source contributions, supporting air-quality planning, and informing policy decisions. However, in order to maintain reasonable computational time and costs, most modeling studies focused on NO_*x*_ chemistry remain at a spatial resolution too coarse to see individual roadway effects. While these setups have generally been able to answer questions about the distribution and sources of NO_*x*_ on state-wide and continental levels, they have generally not been useful for resolving sharp near-source gradients, especially near major roadways. At higher spatial resolutions, there are several additional benefits, including being able to model the local effects of highways, look at neighborhood-scale differences in human health exposure, and validate models with higher-resolution satellite retrievals. While the current highest resolution satellite measurements for NO_2_ from the Tropospheric Monitoring Instrument (TROPOMI) include reprocessed data at a resolution of 3.5 km × 5.5 km,^25^ new instruments such as the Tropospheric Emissions: Monitoring of Pollution (TEMPO) instrument will provide even higher-resolution data on pollutant concentrations in the future.^26^ Moving to a high-resolution inventory is in line with recent improvements in satellite spatial resolution and the increasing need to examine how changes to emissions affect the exposure of distinct communities to air pollutants.

Fuel-based inventory methods provide a complementary perspective to emission model predictions and trends inferred from satellite data, providing critical insights into how emission control technologies have impacted NO_*x*_ concentrations in heavily trafficked urban areas. To gain a better representation of the temporal and spatial patterns of NO_*x*_, this research develops and evaluates a 1.3 km × 1.3 km high-resolution fuel-based inventory for Los Angeles, CA. This study evaluates this inventory as input in the Weather Research and Forecasting with Chemistry (WRF-Chem) model by comparing aircraft measurements taken during the June 2021 RECAP-CA campaign and vertically integrated tropospheric NO_2_ concentrations from the Sentinel-5P/TROPOMI satellite. The resulting spatial and temporal agreement with observational data allows us to understand the individual contributions of NO_*x*_ emission sectors in a major urban area.

## Methods

### Atmospheric Model and Study Domain

We use WRF-Chem (version 4.2.2^27^) to predict meteorological and air-quality-related variables for all of June 2021, using the last 3 days of May as a model spin-up period. The model was applied over two nested domains: (1) all of California and Nevada at 4 km horizontal resolution and (2) southern California at an unusually fine horizontal resolution of 1.3 km. The model includes 50 vertical levels of up to 50 hPa. Initial and boundary conditions for chemical species tracked in the model are from a parent 12 km resolution continental US simulation that used a similar model setup and covered the same period. Initial and boundary conditions for meteorological variables are from the 3 km horizontal resolution High-Resolution Rapid Refresh (HRRR) model, which features hourly assimilation of meteorological variables such as wind and temperature from in-flight commercial aircraft, as well as ground-based radar reflectivity observations of precipitation.^28^ Several meteorological setups were tested and compared with RECAP-CA aircraft wind measurements. Using HRRR gave better model performance for wind speed and direction relative to setups using the North American Mesoscale (NAM) and Rapid Refresh (RAP) models. The chemical mechanism used in this study is a version of the Regional Atmospheric Chemistry Mechanism^29^ with updates to account for key oxygenated volatile chemical product (VCP) emissions (RACM-ESRL-VCP) as specified in Coggon et al.^29,30^ Chemical vertical mixing is enhanced for a low boundary layer height under polluted and fire conditions. Further details of the input data and WRF-Chem model parametrizations are included in Table S1 of the Supporting Information.

### Emission Inventory

Spatially and temporally resolved emissions from on-road vehicles in southern California were estimated and mapped using a modified version of the Fuel-based Inventory for Vehicle Emissions (FIVE).^31,32^ The methodology used to create the FIVE inventory and temporal scaling factors can be found in further detail in Harkins et al.^31^ Emissions are distributed spatially using 2018 data from a national database of traffic counts from the Federal Highway Administration and are scaled to June 2021 using state-wide taxable fuel sales.^33,34^ Comparing the 2018 FHWA traffic counts to state-level traffic statistics, the traffic count data accounts for the majority of fuel use both for gasoline (68%) and for diesel fuel (77%) in California.^35^ The remaining fuel use is mostly on small local roads rather than on major roadways and can thus be adequately distributed using census block-level population data gridded to 1.3 km resolution.^36^ Gridded gasoline and diesel fuel consumption estimates are combined with fuel-specific emission factors (grams of pollutant emitted per kilogram of gasoline or diesel fuel burned) to calculate emissions. Emission factors for each vehicle type are determined based on regression analyses of measurements from roadside remote sensing and highway tunnel studies conducted in California.^7^

The major modifications to the FIVE inventory for this study include moving to 1.3 km resolution and the reapportionment of 22% of the total diesel fuel to a light-/medium-duty diesel vehicle category, separate from the existing gasoline and heavy-duty diesel vehicle categories. These light-/medium-duty diesel vehicles now have higher fuel-based NO_*x*_ emission factors compared to heavy-duty diesel trucks.^7^ Reasons for higher emission factors in this category may vary due to the inclusion of a wide range of vehicle types, but contributing factors include slow fleet turnover of light-duty diesel engines and less deployment of advanced emission control technology (i.e., selective catalytic reduction for NO_*x*_ control) in comparison with heavy-duty diesel trucks. Heavy-duty diesel engines have been a high state-wide priority in efforts to accelerate replacement of older (pre-2010) engines with newer and lower-emitting engines.^37^ Diurnal and day-of-week variations in vehicular emissions are based on weigh-in-motion traffic count data, with separate temporal variation profiles for light- and heavy-duty vehicles.^32^ We assume that the spatial distribution of light- and medium-duty diesel truck traffic matches that of light-duty gasoline vehicles and use the activity profile for heavy-duty trucks to specify temporal variations. In comparison to the existing FIVE inventory, this reapportionment of diesel fuel to light-/medium-duty vehicles results in higher on-road NO_*x*_ emissions overall, especially on weekdays, and more emissions being attributed to light-duty vehicle spatial patterns.

In addition to on-road vehicle emissions, the emission inventory used in this study also includes fuel-based emissions from off-road engines used in agricultural and construction equipment.^31,38,39^ Also included are emissions from oil and natural gas production,^40^ power plants based on Continuous Emission Monitoring System data,^41^ and other point and area sources from the 2017 National Emission Inventory scaled to 2021 based on activity factors.^31,42^ Ocean-going vessel emissions and emissions from all sources in Mexico were from the Copernicus Atmosphere Monitoring Service (CAMS) inventory.^43^ Biogenic emissions are based on the Biogenic Emissions Inventory System (BEIS) v3.14 model.^44,45^ Consistent with previous modeling work in Los Angeles,^46^ BEIS emissions for isoprene and monoterpenes from the urban land cover type were updated based on Scott and Benjamin.^47^

### Aircraft and Surface Monitor Data

Model predictions were compared to vertical profile NO_2_ measured during aircraft flights over the Los Angeles basin that took place during the summer of 2021 as part of the RECAP-CA (Re-Evaluating the Chemistry of Air Pollutants in CAlifornia) field campaign.^48,49^ Relevant flights occurred at midday hours on three weekend days (June 6, 12, and 19) and six weekdays (June 1, 4, 10, 11, 18, and 21). NO_*x*_ was measured at 5 Hz using a thermal dissociation laser-induced fluorescence (TD-LIF) instrument, and a detailed overview of the instrument can be found in Thornton et al. and Day et al.^50,51^ Instrument calibration details and methodology used during this campaign can be found in Zhu et al.^49^ Here, we report only data from the stacked racetrack patterns (see Figure S1), where the plane flew 4–6 different altitudes’ layer stacked on top of one another within the planetary boundary, which were designed to measure vertical concentration profiles. The aircraft data used here were split into flights that occurred closer to the coast (west/central LA) and at locations further inland (east basin) where temperatures were higher. Only altitude bins with greater than five observation points were used in the comparison. The aircraft data were matched with corresponding model predictions using a nearest neighbor method, averaging the aircraft data every 30 s and pulling comparison data for the nearest model grid point.

In addition to verifying the vertical profiles against aircraft observations, the South Coast Air Quality Management District (AQMD) monitoring network was used for comparison to verify the diurnal patterns of NO_2_ in the model.^46^ The NO_2_ data for 24 sites located in the South Coast Air Basin were averaged for each hour of the day on weekdays and weekends. The quantity of NO_2_* (NO_2_* = NO_2_ + PAN + alkyl nitrates + HONO + 2*N_2_O_5_) was used from the model for comparison to the measured NO_2_ due to the conversion of other nitrogenous species in addition to NO_2_ by the molybdenum converter installed within standard chemiluminescent NO_*x*_ analyzers.^52,53^

### Satellite Observations

TROPOMI satellite measurements are used in this study to evaluate tropospheric column-integrated model predictions of NO_2_ concentrations. We use the latest NO_2_ product reprocessed to a spatial resolution of 3.5 km × 5.5 km from the Sentinel-5P Products Algorithm Laboratory (SP5-PAL) for June 2021.^25,54^ We used this reprocessed data product to create average June tropospheric NO_2_ columns using methods described by Li et al., as well as separate weekday and weekend average tropospheric NO_2_ columns for the study period.^55^ Pixels with cloud cover, snow, or ice, or with otherwise problematic retrievals, are filtered out to reduce uncertainties by using only data where the quality flag ≥0.75.^56^ NO_2_ vertical profiles require an assumption for the air mass factor (AMF), which may affect the ability to compare tropospheric NO_2_ columns between the model and satellite data. In order to eliminate biases introduced by a priori profile assumptions, the NO_2_ vertical profiles used in this study are calculated using averaging kernels and the AMF was calculated from WRF-Chem, rather than using the AMF derived from the a priori TM5 model.^57^ This approach has been shown to increase the satellite NO_2_ concentrations over urban areas by 20% on average.^55^ Even accounting for these improvements, there is still an expectation of a low bias of −20% in TROPOMI tropospheric NO_2_ columns over polluted cities in comparison to observations based on studies compared to ground-based Pandora measurements.^54,55,58^

## Results and Discussion

**Figure 1:**
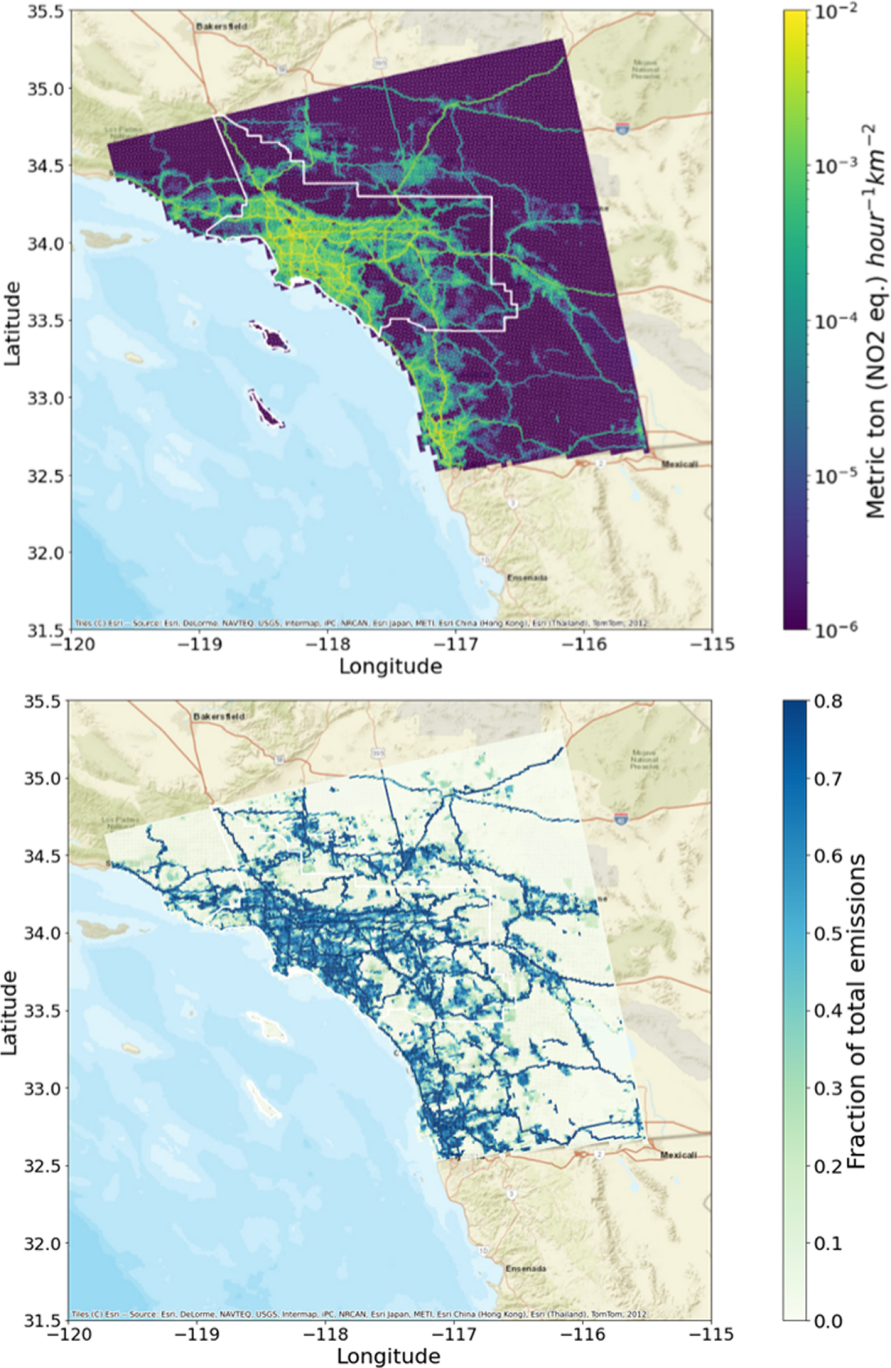
Spatial distribution of average 2021 weekday NO_*x*_ emission rates from on-road vehicles in southern California (top) and the fraction of total emissions from on-road vehicles (bottom), mapped at a 1.3 km horizontal resolution. The South Coast Air Basin is outlined..

Figure 1 shows the spatial distribution of NO_*x*_ emissions from on-road vehicles for the 1.3 km resolution southern California modeling domain. NO_*x*_ emissions are the highest in the urban center, with lower emissions in sparsely populated rural and mountainous areas. This pattern follows from the emissions mapping methodology, which relies on vehicle traffic counts that indicate high traffic densities within urban areas. Sharp spatial gradients in vehicle emissions near major highways are clearly apparent in the top panel of Figure 1, not only in rural areas but also within densely populated urban areas. While high NO_*x*_ emissions in and around downtown LA are mostly due to high volumes of light-duty vehicle traffic, elsewhere along major highways such as I-5 (running to the northwest of LA) and I-15 (running to the northeast of LA toward Las Vegas), the majority of the NO_*x*_ emissions are due to heavy-duty diesel trucks. Even though emissions from on-road vehicles decrease on weekends primarily due to the steep weekend decrease in diesel vehicle activity, vehicles overall are still the dominant source of NO_*x*_ emissions, responsible for 52% of the NO_*x*_ on weekends versus 62% of the NO_*x*_ on weekdays. The fraction of the total emissions from the on-road sector on weekdays can be seen in the bottom panel of Figure 1.

More details concerning NO_*x*_ emissions from on-road vehicles are presented in Table 1. We estimate that average daily NO_*x*_ emissions for the South Coast Air Basin were 198 ± 69 t/day during our June 2021 study period, which is 1.6 ± 0.5 times the corresponding summer-season estimate from the California EMFAC model. Our estimates of NO_*x*_ emissions are comparable for heavy-duty diesel and higher for gasoline and light-/medium-duty diesel categories, with the largest relative difference (a ratio of 2.8 ± 0.6) for light- and medium-duty diesel vehicles. As shown in Table 1, on-road vehicle emissions are lower on weekends than on weekdays, as expected, due mainly to the decreased activity and emissions from diesel trucks. The overall decrease in vehicular NO_*x*_ emissions on weekends is about 100 t/day, which is a 43% reduction relative to baseline weekday conditions. A potential cause of the lower emissions in the EMFAC model in comparison to the fuel-based inventory is in how emission factor trends are derived. The modified FIVE inventory used in this study uses on-road remote-sensing data trends, resulting in emission factors that are higher overall and are decreasing more slowly in comparison to laboratory-measured emission factors used in other inventories. The large sample size of remote-sensing data is better equipped to capture the effects of individual vehicles with ineffective or nonfunctioning emission control systems. While the remote-sensing studies capture driving conditions that reflect typical vehicle activity patterns, they do not capture start-up and idling conditions, leaving room for actual average emission factors to be even higher. In the case of heavy-duty vehicles, selective catalytic reduction (SCR) systems do not operate effectively at low temperatures, leading to potential underestimates of mobile source emissions in urban areas when idealized SCR performance is assumed.

**Table 1:** Estimates of NO_*x*_ Emissions from On-Road Vehicles in Southern California^a^

	vehicle category			
	gasoline	LD + MD diesel^b^	HD diesel^b^	on-road total
fuel burned (t/day)	41,763 ± 2506	2295 ± 298	6015 ± 782	
EF NO_*x*_ (g/kg)	2.0 ± 0.6	20.2 ± 3.4	9.2 ± 4.6	
avg emissions (t/day)	87 ± 27	49 ± 10	62 ± 32	198 ± 69
WD^c^ emissions (t/day)	89	60	76	224
WE^c^ emissions (t/day)	82	20	26	127
EMFAC (t/day)	59	17	48	124
fuel-based/EMFAC^d^	1.5 ± 0.4	2.8 ± 0.6	1.3 ± 0.7	1.6 ± 0.5

a Emission estimates for the South Coast Air Basin, including Los Angeles, Orange County, and portions of Riverside and San Bernardino Counties.

b Diesel vehicle weight categories are light-, medium-, and heavy-duty (LD, MD, and HD).

c Emissions for average weekday (WD) and weekend (WE) conditions.

d Ratio of fuel-based emission inventory (weighted average of WD and WE values) from this study with corresponding estimates for summer 2021 from the most current version of the EMFAC model.^59^

**Figure 2:**
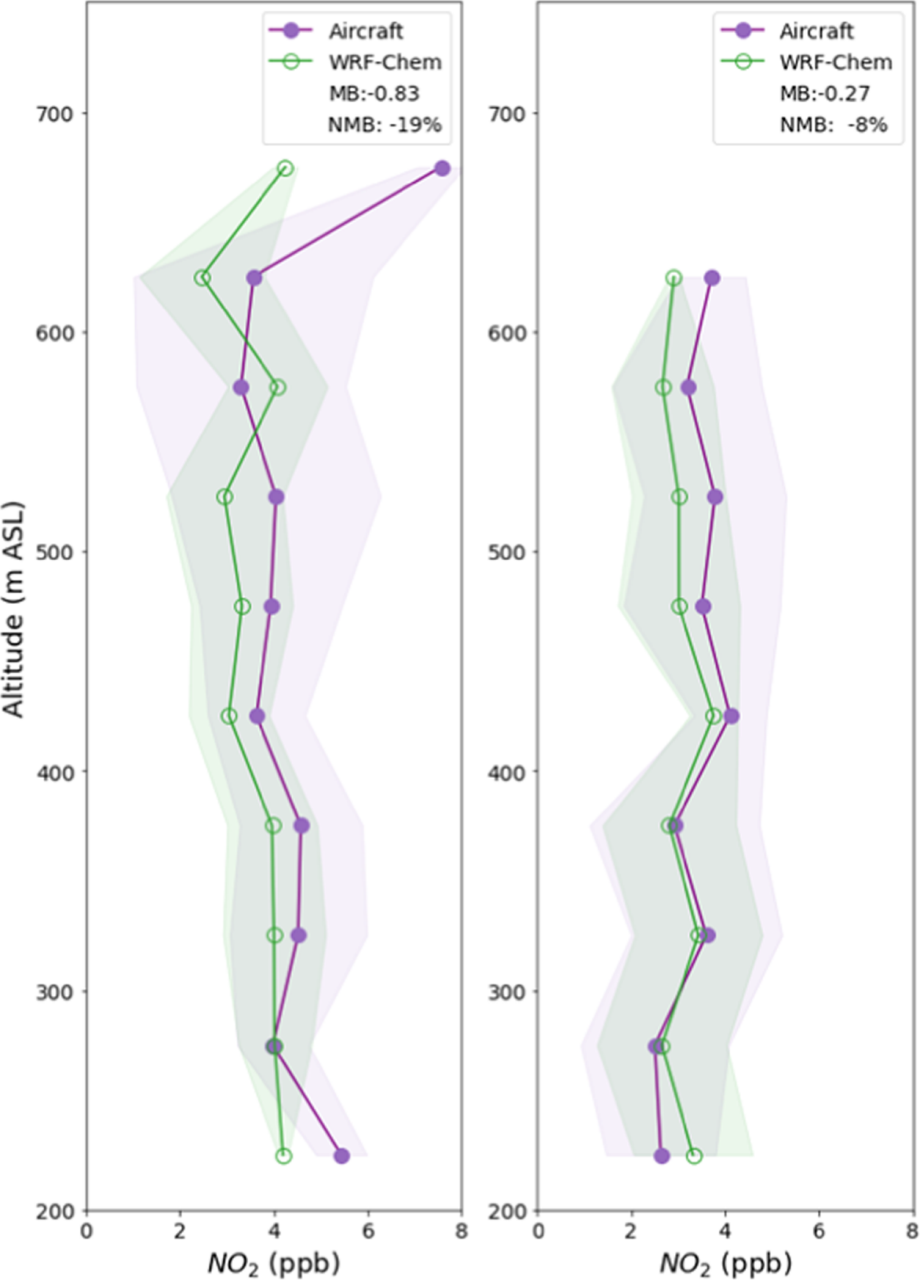
Vertical profiles of measured (RECAP flights) and modeled NO_2_ concentrations over Western (left panel) and Eastern Los Angeles (right panel), binned over 100 m intervals. Shaded bands indicate 1 standard deviation from the mean values.

Figure 2 shows modeled and measured NO_2_ concentrations as a function of altitude above sea level; values are binned within 100 m intervals of altitude. The plots are separated into two regions, Western LA and Eastern LA, in order to eliminate spatial biases. Flight tracks showing the specific locations of these measurements are shown in Figure S1. Normalized mean biases for NO_2_ are −19 and −8% for the western and eastern portions of Los Angeles, respectively. Vertical profiles are in good agreement at most altitudes, especially below 600 m. In Eastern LA, the NO_2_ concentrations are closer to the observations near the surface and diverge slightly with an increasing altitude. In Western LA, we see that generally the model and observation of NO_2_ match very closely, especially between 250 and 600 m. Despite slight underestimation in the model compared to the aircraft observations overall, these results indicate that the vertical representation of NO_2_ in the model closely resembles the NO_2_ measured during the field campaign during the same period. An accurate vertical profile in the model is critical to the calculation of tropospheric NO_2_ columns from the satellite, as the AMF used in the calculation is revised to use the NO_2_ vertical profile shape from the WRF-Chem simulation and averaging kernels.

The comparison of the diurnal patterns between the model and AQMD surface observations for the June 2021 study period is shown in Figure S2. This comparison suggests that the diurnal variations in modeled NO_2_* are consistent with observations, but there is some negative bias in the modeled NO_2_* concentrations during nighttime hours on the weekends. The analysis performed at a 4 km resolution yielded the same conclusions.

**Figure 3:**
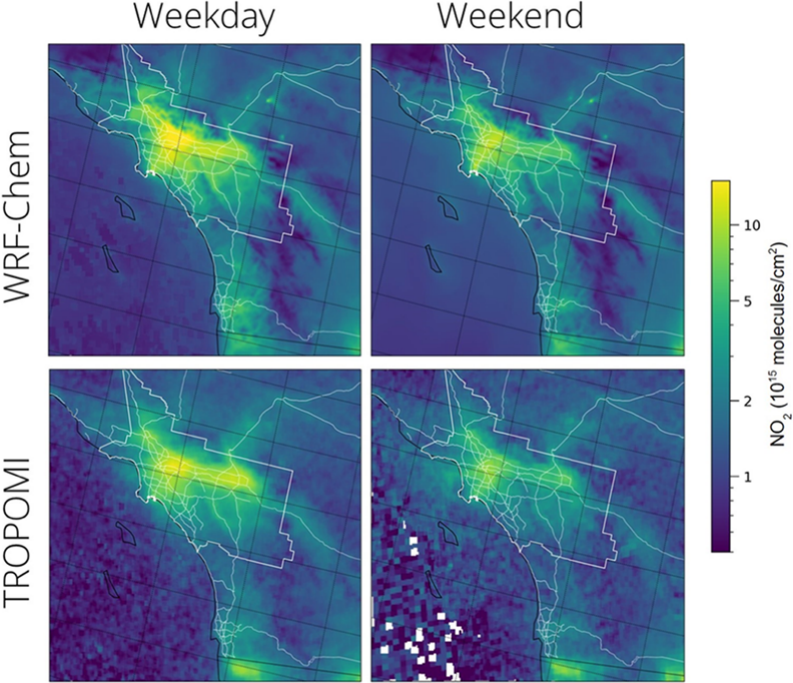
Average tropospheric NO_2_ columns over southern California for June 2021 were predicted using the WRF-Chem model (top row) with comparisons to TROPOMI satellite data (bottom row). Separate results are shown for weekdays (left panels) and weekends (right panels). The boundaries of the South Coast Air Basin and major highways are shown in white.

Figure 3 compares spatial distributions of modeled (WRF-Chem) tropospheric column-integrated NO_2_ concentrations with the corresponding TROPOMI satellite-derived values. Both model and satellite data show high weekday NO_2_ columns over downtown LA extending south toward Long Beach as well as extending further inland to the east as far as San Bernardino. In both cases, there is significantly lower NO_2_ on the weekends, especially apparent within the South Coast Air Basin along the corridors east and south of downtown LA. Elevated NO_2_ concentrations due to traffic along major highways running through rural areas are captured well, notably on Interstate highways I-5 (heading north toward San Francisco and Sacramento) and I-15 (heading northeast toward Las Vegas) (see Figure S3 of the Supporting Information for interstate locations). The main difference between modeled and observed NO_2_ in the southern California domain is that, overall, WRF-Chem has higher NO_2_ concentrations in the downtown region, with maximum NO_2_ concentrations slightly offset to the northeast compared to what the TROPOMI satellite observations show (Figure S4). These differences are slightly emphasized on weekdays in comparison to weekends.

**Figure 4:**
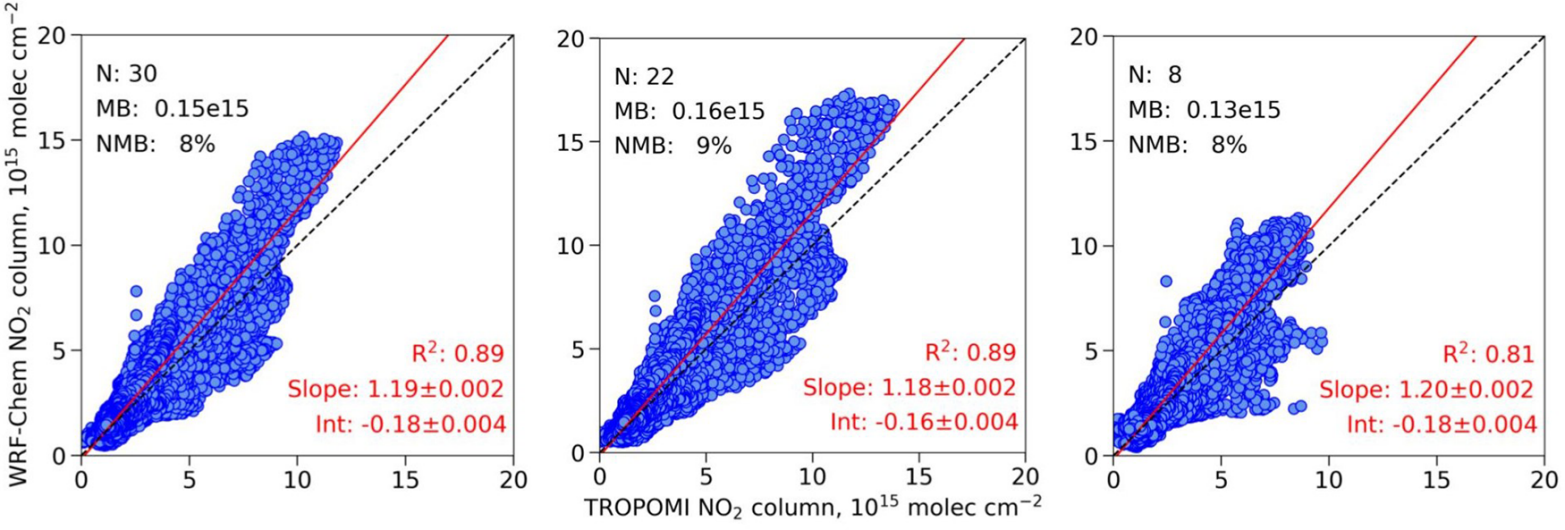
Orthogonal distance regression between modeled and satellite-derived tropospheric NO_2_ columns over southern California in June 2021 for all days (left panel), weekdays only (middle panel), and weekends only (right panel).

Figure 4 shows scatterplots of modeled tropospheric NO_2_ columns versus corresponding satellite-derived values. The lower NO_2_ values that prevail on weekends are apparent in the more limited range of the data in the rightmost panel of Figure 4. The regression-derived coefficients are similar for all three plots, with near-zero intercepts and slopes of 1.18–1.20. The model explains a high fraction of the observed variance in NO_2_ columns, although the value of R^2^ decreases somewhat from 0.89 on weekdays to 0.81 on weekends. Mean normalized bias for the model relative to satellite data is +8% for all days in June, with similar values for the subsets of weekdays only and weekend days only. The finding of slight overestimation in the model compared to the satellite data is in contrast with the finding of slight underestimation compared to the aircraft observations. The differences between the model and the satellite are all within the range of and directionally consistent with findings of previous studies that suggest a negative bias of ∼20% in satellite-derived observations of NO_2_ columns in urban areas.^47^

The direct comparison between model and satellite NO_2_ columns at 4 km resolution over the larger California domain can be seen in Figure S5 of the Supporting Information. Model performance for our study period at a 4 km resolution for California is comparable to model performance at a 1.3 km resolution for southern California within the same domain, as shown in Figure S6 of the Supporting Information. At 4 km resolution, we see an *R*^2^ of 0.83 with a slope of 1.14 for the entire California/Nevada domain and an *R*^2^ of 0.91 and a slope of 1.09 for the southern California domain. While the results have similar statistics, the 1.3 km output has the added benefit of resolving sharp gradients in NO_2_ over major cities and highways, which is in line with what can be resolved by the satellite.

**Figure 5:**
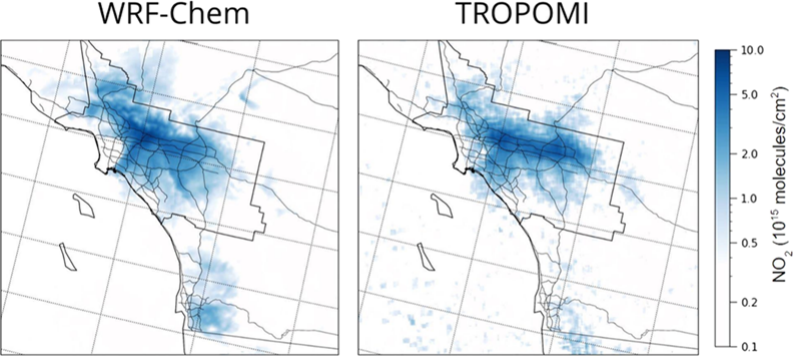
Weekday–weekend difference plots for tropospheric NO_2_ columns for the WRF-Chem model and TROPOMI satellite data.

To further evaluate the emission inventory, we turn to a more in-depth consideration of weekday–weekend differences in the NO_2_ columns. Figure 5 shows weekday–weekend difference plots of NO_2_ columns for the model and for the satellite data. The spatial patterns of areas showing large weekend decreases in these plots are similar. The model shows larger weekend NO_2_ decreases northwest of downtown along the I-5 Interstate highway through the San Fernando Valley, while the satellite data indicates larger weekend NO_2_ decreases further inland, extending to the east as far as San Bernardino. The traffic count data underlying the fuel-based inventory used in this study are for 2018 and may not adequately reflect expansions in warehousing, truck traffic, and associated NO_*x*_ emissions that have recently occurred in inland portions of the LA basin.^60,61^

An additional emission sensitivity case was considered in a model run with all on-road vehicle emissions zeroed out completely. Emissions from other source categories were left unchanged. As shown in Figure S7, the spatial pattern in the resulting weekday–weekend difference plot for this case without the on-road sector does not match either the observed spatial distribution or the magnitude of weekend NO_2_ decreases shown in Figure 5. We conclude that locations that exhibit large weekend NO_2_ decreases (i.e., from Downtown LA east to San Bernardino) are those where on-road vehicle emissions make large absolute and relative contributions to tropospheric NO_2_ columns.

Current emission inventories used for air-quality planning purposes suggest that on-road vehicle emissions are no longer the dominant source of NO_*x*_ emissions in southern California. In contrast, as shown in Table 2, the emission inventory in this study has 25% higher NO_*x*_ emissions overall when compared to the planning inventory, with the dominant contribution coming from on-road sources. More specifically, the present study includes higher emissions from on-road vehicles (198 ± 69 vs 103 tons/day) that account for 66% of the anthropogenic NO_*x*_ emissions. The planning inventory assigns greater importance to area and off-road mobile sources, which together account for a higher fraction (49 vs 26%) of total NO_*x*_ emissions compared to the present study.

**Table 2:** Comparison of NO_*x*_ Emission Inventories for the South Coast Air Basin

	fuel-based inventory June 2021		south coast air-quality management district, summer 2022^a^	
source sector	NO_*x*_ (t/day)	fraction of total	NO_*x*_ (t/day)	fraction of total
on-road	198 ± 69	66%	103	43%
area + off-road	79	26%	116	49%
point	23	8%	20	8%
total	300		239	

a Ocean-going vessels removed to match included sectors of fuel-based inventory.

Air-quality model results, satellite observations, and their corresponding weekday–weekend differences are consistent with a larger-than-expected on-road vehicular source of NO_*x*_ emissions in southern California. Major efforts to update fleets to newer engine models with advanced emission control systems have improved heavy-duty diesel, and values from the EMFAC model are consistent with and within the uncertainty of those from the fuel-based inventory evaluated here. Still, actual emissions from heavy-duty diesel may be even higher than estimated due to ineffective NO_*x*_ control during idling and low load driving conditions. Total NO_*x*_ emission estimates are higher in this study for on-road vehicles in comparison to the EMFAC inventory owing to higher emissions from gasoline vehicles and light- and medium-duty diesel vehicles. Contributing factors to these differences may include slow absolute progress in reducing fleet-average NO_*x*_ emission factors for gasoline vehicles since 2010 (see Figure 2 in Yu et al., 2021) and slow fleet turnover with relatively high in-use NO_*x*_ emission factors for light- and medium-duty diesel trucks.

## Supporting Information Available

The emission inventory used in this study is available at the following URL: https://csl.noaa.gov/groups/csl7/measurements/2021sunvex/emissions/. The Supporting Information is available free of charge at https://pubs.acs.org/doi/10.1021/acs.est.3c06158.

Summary of the WRF-Chem model setup and input data, flight paths of aircraft, diurnal pattern comparison with AQMD sensors, key interstate freeways in the Los Angeles domain, absolute difference plots between model and satellite, average tropospheric NO_2_ columns at 4 km resolution, orthogonal distance regression between model and satellite NO_2_ at 4 km resolution for the California/Nevada domain and the southern California domain, weekday–weekend difference of the on-road sector and combined off-road, area, and point sectors (PDF)

## References

[ref1] Jerrett M.; Burnett R. T.; Pope C. A.; Ito K.; Thurston G.; Krewski D.; Shi Y.; Calle E.; Thun M. Long-Term Ozone Exposure and Mortality. N. Engl. J. Med. 2009, 360 (11), 1085–1095. 10.1056/NEJMoa0803894. 19279340 PMC4105969

[ref2] Burnett R.; Chen H.; Szyszkowicz M.; Fann N.; Hubbell B.; Pope C. A.; Apte J. S.; Brauer M.; Cohen A.; Weichenthal S.; Coggins J.; Di Q.; Brunekreef B.; Frostad J.; Lim S. S.; Kan H.; Walker K. D.; Thurston G. D.; Hayes R. B.; Lim C. C.; Turner M. C.; Jerrett M.; Krewski D.; Gapstur S. M.; Diver W. R.; Ostro B.; Goldberg D.; Crouse D. L.; Martin R. V.; Peters P.; Pinault L.; Tjepkema M.; van Donkelaar A.; Villeneuve P. J.; Miller A. B.; Yin P.; Zhou M.; Wang L.; Janssen N. A. H.; Marra M.; Atkinson R. W.; Tsang H.; Quoc Thach T.; Cannon J. B.; Allen R. T.; Hart J. E.; Laden F.; Cesaroni G.; Forastiere F.; Weinmayr G.; Jaensch A.; Nagel G.; Concin H.; Spadaro J. V. Global Estimates of Mortality Associated with Long-Term Exposure to Outdoor Fine Particulate Matter. Proc. Natl. Acad. Sci. U.S.A. 2018, 115 (38), 9592–9597. 10.1073/pnas.1803222115. 30181279 PMC6156628

[ref3] Kim K.-H.; Kabir E.; Kabir S. A Review on the Human Health Impact of Airborne Particulate Matter. Environ. Int. 2015, 74, 136–143. 10.1016/j.envint.2014.10.005. 25454230

[ref4] Seinfeld J. H.; Pandis S. N. Atmospheric Chemistry and Physics: From Air Pollution to Climate Change; Wiley-Interscience: Hoboken, N.J, 2006; pp 44–45.

[ref5] Dallmann T. R.; Harley R. A. Evaluation of Mobile Source Emission Trends in the United States. J. Geophys. Res.: Atmos. 2010, 115 (D14), D14305 10.1029/2010JD013862.

[ref6] McDonald B. C.; Dallmann T. R.; Martin E. W.; Harley R. A. Long-Term Trends in Nitrogen Oxide Emissions from Motor Vehicles at National, State, and Air Basin Scales. J. Geophys. Res.: Atmos. 2012, 117 (D21), D00V18 10.1029/2012JD018304.

[ref7] Yu K. A.; McDonald B. C.; Harley R. A. Evaluation of Nitrogen Oxide Emission Inventories and Trends for On-Road Gasoline and Diesel Vehicles. Environ. Sci. Technol. 2021, 55 (10), 6655–6664. 10.1021/acs.est.1c00586. 33951912

[ref8] Yanowitz J.; McCormick R. L.; Graboski M. S. In-Use Emissions from Heavy-Duty Diesel Vehicles. Environ. Sci. Technol. 2000, 34 (5), 729–740. 10.1021/es990903w. 11827062

[ref9] Preble C. V.; Dallmann T. R.; Kreisberg N. M.; Hering S. V.; Harley R. A.; Kirchstetter T. W. Effects of Particle Filters and Selective Catalytic Reduction on Heavy-Duty Diesel Drayage Truck Emissions at the Port of Oakland. Environ. Sci. Technol. 2015, 49 (14), 8864–8871. 10.1021/acs.est.5b01117. 26083075

[ref10] Preble C. V.; Harley R. A.; Kirchstetter T. W. Control Technology-Driven Changes to In-Use Heavy-Duty Diesel Truck Emissions of Nitrogenous Species and Related Environmental Impacts. Environ. Sci. Technol. 2019, 53 (24), 14568–14576. 10.1021/acs.est.9b04763. 31686501

[ref11] Bishop G. A.; Hottor-Raguindin R.; Stedman D. H.; McClintock P.; Theobald E.; Johnson J. D.; Lee D.-W.; Zietsman J.; Misra C. On-Road Heavy-Duty Vehicle Emissions Monitoring System. Environ. Sci. Technol. 2015, 49 (3), 1639–1645. 10.1021/es505534e. 25606715

[ref12] Haugen M. J.; Bishop G. A. Repeat Fuel Specific Emission Measurements on Two California Heavy-Duty Truck Fleets. Environ. Sci. Technol. 2017, 51 (7), 4100–4107. 10.1021/acs.est.6b06172. 28290679

[ref13] Haugen M. J.; Bishop G. A. Long-Term Fuel-Specific NOx and Particle Emission Trends for In-Use Heavy-Duty Vehicles in California. Environ. Sci. Technol. 2018, 52 (10), 6070–6076. 10.1021/acs.est.8b00621. 29692175

[ref14] Thiruvengadam A.; Besch M. C.; Thiruvengadam P.; Pradhan S.; Carder D.; Kappanna H.; Gautam M.; Oshinuga A.; Hogo H.; Miyasato M. Emission Rates of Regulated Pollutants from Current Technology Heavy-Duty Diesel and Natural Gas Goods Movement Vehicles. Environ. Sci. Technol. 2015, 49 (8), 5236–5244. 10.1021/acs.est.5b00943. 25826745

[ref15] Anderson D. C.; Loughner C. P.; Diskin G.; Weinheimer A.; Canty T. P.; Salawitch R. J.; Worden H. M.; Fried A.; Mikoviny T.; Wisthaler A.; Dickerson R. R. Measured and Modeled CO and NOy in DISCOVER-AQ: An Evaluation of Emissions and Chemistry over the Eastern US. Atmos. Environ. 2014, 96, 78–87. 10.1016/j.atmosenv.2014.07.004.

[ref16] Travis K. R.; Jacob D. J.; Fisher J. A.; Kim P. S.; Marais E. A.; Zhu L.; Yu K.; Miller C. C.; Yantosca R. M.; Sulprizio M. P.; Thompson A. M.; Wennberg P. O.; Crounse J. D.; St Clair J. M.; Cohen R. C.; Laughner J. L.; Dibb J. E.; Hall S. R.; Ullmann K.; Wolfe G. M.; Pollack I. B.; Peischl J.; Neuman J. A.; Zhou X. Why Do Models Overestimate Surface Ozone in the Southeast United States?. Atmos. Chem. Phys. 2016, 16 (21), 13561–13577. 10.5194/acp-16-13561-2016. 29619045 PMC5880041

[ref17] McDonald B. C.; McKeen S. A.; Cui Y. Y.; Ahmadov R.; Kim S.-W.; Frost G. J.; Pollack I. B.; Peischl J.; Ryerson T. B.; Holloway J. S.; Graus M.; Warneke C.; Gilman J. B.; de Gouw J. A.; Kaiser J.; Keutsch F. N.; Hanisco T. F.; Wolfe G. M.; Trainer M. Modeling Ozone in the Eastern U.S. Using a Fuel-Based Mobile Source Emissions Inventory. Environ. Sci. Technol. 2018, 52 (13), 7360–7370. 10.1021/acs.est.8b00778. 29870662

[ref18] Jiang Z.; McDonald B. C.; Worden H.; Worden J. R.; Miyazaki K.; Qu Z.; Henze D. K.; Jones D. B. A.; Arellano A. F.; Fischer E. V.; Zhu L.; Boersma K. F. Unexpected Slowdown of US Pollutant Emission Reduction in the Past Decade. Proc. Natl. Acad. Sci. U.S.A. 2018, 115 (20), 5099–5104. 10.1073/pnas.1801191115. 29712822 PMC5960319

[ref19] Gaudel A.; Cooper O. R.; Chang K.-L.; Bourgeois I.; Ziemke J. R.; Strode S. A.; Oman L. D.; Sellitto P.; Nédélec P.; Blot R.; Thouret V.; Granier C. Aircraft Observations since the 1990s Reveal Increases of Tropospheric Ozone at Multiple Locations across the Northern Hemisphere. Sci. Adv. 2020, 6 (34), eaba8272 10.1126/sciadv.aba8272. 32937364 PMC7442356

[ref20] U.S. Environmental Protection Agency. Ozone Trends, Washington D.C., May 4, 2016. https://www.epa.gov/air-trends/ozone-trends (accessed June 05, 2023).

[ref21] Nussbaumer C. M.; Cohen R. C. The Role of Temperature and NOx in Ozone Trends in the Los Angeles Basin. Environ. Sci. Technol. 2020, 54 (24), 15652–15659. 10.1021/acs.est.0c04910. 33274926

[ref22] Laughner J. L.; Cohen R. C. Direct Observation of Changing NOx Lifetime in North American Cities. Science 2019, 366 (6466), 723–727. 10.1126/science.aax6832. 31699933 PMC7301961

[ref23] Port of Los Angeles. Facts and Figures. https://www.portoflosangeles.org/business/statistics/facts-and-figures (accessed May 31, 2023).

[ref24] Environmental Protection Agency. Clean Air Plans; 2015 8-h Ozone Nonattainment Area Requirements; Clean Fuels or Advanced Control Technology for Boilers; San Joaquin Valley and Los Angeles-South Coast Air Basin, California. 2023. https://www.federalregister.gov/d/2023-01504 (accessed May 31, 2023).

[ref25] Veefkind J. P.; Aben I.; McMullan K.; Förster H.; de Vries J.; Otter G.; Claas J.; Eskes H. J.; de Haan J. F.; Kleipool Q.; van Weele M.; Hasekamp O.; Hoogeveen R.; Landgraf J.; Snel R.; Tol P.; Ingmann P.; Voors R.; Kruizinga B.; Vink R.; Visser H.; Levelt P. F. TROPOMI on the ESA Sentinel-5 Precursor: A GMES Mission for Global Observations of the Atmospheric Composition for Climate, Air Quality and Ozone Layer Applications. Remote Sens. Environ. 2012, 120, 70–83. 10.1016/j.rse.2011.09.027.

[ref26] Zoogman P.; Liu X.; Suleiman R. M.; Pennington W. F.; Flittner D. E.; Al-Saadi J. A.; Hilton B. B.; Nicks D. K.; Newchurch M. J.; Carr J. L.; Janz S. J.; Andraschko M. R.; Arola A.; Baker B. D.; Canova B. P.; Chan Miller C.; Cohen R. C.; Davis J. E.; Dussault M. E.; Edwards D. P.; Fishman J.; Ghulam A.; Abad G. G.; Grutter M.; Herman J. R.; Houck J.; Jacob D. J.; Joiner J.; Kerridge B. J.; Kim J.; Krotkov N. A.; Lamsal L.; Li C.; Lindfors A.; Martin R. V.; McElroy C. T.; McLinden C.; Natraj V.; Neil D. O.; Nowlan C. R.; O’Sullivan E. J.; Palmer P. I.; Pierce R. B.; Pippin M. R.; Saiz-Lopez A.; Spurr R. J. D.; Szykman J. J.; Torres O.; Veefkind J. P.; Veihelmann B.; Wang H.; Wang J.; Chance K. Tropospheric Emissions: Monitoring of Pollution (TEMPO). J. Quant. Spectrosc. Radiat. Transfer 2017, 186, 17–39. 10.1016/j.jqsrt.2016.05.008. PMC7430511 32817995

[ref27] Grell G. A.; Peckham S. E.; Schmitz R.; McKeen S. A.; Frost G.; Skamarock W. C.; Eder B. Fully Coupled “Online” Chemistry within the WRF Model. Atmos. Environ. 2005, 39 (37), 6957–6975. 10.1016/j.atmosenv.2005.04.027.

[ref28] Benjamin S. G.; Weygandt S. S.; Brown J. M.; Hu M.; Alexander C. R.; Smirnova T. G.; Olson J. B.; James E. P.; Dowell D. C.; Grell G. A.; Lin H.; Peckham S. E.; Smith T. L.; Moninger W. R.; Kenyon J. S.; Manikin G. S. A North American Hourly Assimilation and Model Forecast Cycle: The Rapid Refresh. Mon. Weather Rev. 2016, 144 (4), 1669–1694. 10.1175/MWR-D-15-0242.1.

[ref29] Stockwell W. R.; Kirchner F.; Kuhn M.; Seefeld S. A New Mechanism for Regional Atmospheric Chemistry Modeling. J. Geophys. Res.: Atmos. 1997, 102 (D22), 25847–25879. 10.1029/97JD00849.

[ref30] Coggon M. M.; Gkatzelis G. I.; McDonald B. C.; Gilman J. B.; Schwantes R. H.; Abuhassan N.; Aikin K. C.; Arend M. F.; Berkoff T. A.; Brown S. S.; Campos T. L.; Dickerson R. R.; Gronoff G.; Hurley J. F.; Isaacman-VanWertz G.; Koss A. R.; Li M.; McKeen S. A.; Moshary F.; Peischl J.; Pospisilova V.; Ren X.; Wilson A.; Wu Y.; Trainer M.; Warneke C. Volatile Chemical Product Emissions Enhance Ozone and Modulate Urban Chemistry. Proc. Natl. Acad. Sci. U.S.A. 2021, 118 (32), e2026653118 10.1073/pnas.2026653118. 34341119 PMC8364211

[ref31] Harkins C.; McDonald B. C.; Henze D. K.; Wiedinmyer C. A Fuel-Based Method for Updating Mobile Source Emissions during the COVID-19 Pandemic. Environ. Res. Lett. 2021, 16 (6), 065018 10.1088/1748-9326/ac0660.

[ref32] McDonald B. C.; McBride Z. C.; Martin E. W.; Harley R. A. High-Resolution Mapping of Motor Vehicle Carbon Dioxide Emissions. J. Geophys. Res.: Atmos. 2014, 119 (9), 5283–5298. 10.1002/2013JD021219.

[ref33] Federal Highway Administration. 2018 HPMS Public Release. https://www.fhwa.dot.gov/policyinformation/hpms/shapefiles.cfm (accessed May 31, 2023).

[ref34] California Department of Tax and Fee Administration. Motor Fuel 10 Year Report and Taxable Diesel Gallons 10 Year Report. https://www.cdtfa.ca.gov/taxes-and-fees/spftrpts.htm (accessed May 31, 2023).

[ref35] Federal Highway Administration. Highway Statistics Reports 1990–2018 Table MF-21 https://www.fhwa.dot.gov/policyinformation/statistics.cfm (accessed May 31, 2023).

[ref36] U.S. Census Bureau. Special Release- Census Blocks with Population and Housing Counts. https://www.census.gov/geographies/mapping-files/2010/geo/tiger-line-file.html (accessed May 31, 2023).

[ref37] US Environmental Protection Agency. Emission Standards for 2004 and Later Model Year Diesel Heavy-Duty Engines and Vehicles, Code of Federal Regulations 2012. 40 CFR § 86.004–11.

[ref38] Kean A. J.; Sawyer R. F.; Harley R. A. A Fuel-Based Assessment of Off-Road Diesel Engine Emissions. J. Air Waste Manage. Assoc. 2000, 50 (11), 1929–1939. 10.1080/10473289.2000.10464233. 11111337

[ref39] Energy Information Administration. Fuel Oil and Kerosene Sales. https://www.eia.gov/petroleum/fueloilkerosene/ (accessed May 31, 2023).

[ref40] Francoeur C. B.; McDonald B. C.; Gilman J. B.; Zarzana K. J.; Dix B.; Brown S. S.; de Gouw J. A.; Frost G. J.; Li M.; McKeen S. A.; Peischl J.; Pollack I. B.; Ryerson T. B.; Thompson C.; Warneke C.; Trainer M. Quantifying Methane and Ozone Precursor Emissions from Oil and Gas Production Regions across the Contiguous US. Environ. Sci. Technol. 2021, 55 (13), 9129–9139. 10.1021/acs.est.0c07352. 34161066

[ref41] U.S. Environmental Protection Agency. Continuous Emission Monitoring Systems. https://www.epa.gov/emc/emc-continuous-emission-monitoring-systems (accessed May 31, 2023).

[ref42] U.S. Environmental Protection Agency. National Emissions Inventory (NEI) 2017, April 2020 Version. https://gispub.epa.gov/neireport/2017/ (accessed May 31, 2023).

[ref43] Granier C.; Darras S.; Denier van der Gon H.; Doubalova J.; Elguindi N.; Galle B.; Gauss M.; Guevara M.; Jalkanen J.-P.; Kuenen J.; Liousse C.; Quack B.; Simpson D.; Sindelarova K. The Copernicus Atmosphere Monitoring Service Global and Regional Emissions, (April 2019 Version). 10.24380/D0BN-KX16.

[ref44] Pierce T. E.; Geron C. D.; Kinnee E.; Vukovich J. Integration of the Biogenic Emissions Inventory System (BEIS3) into the Community Multiscale Air Quality Modeling System, 25th Conference on Agricultural and Forest Meteorology: Norfolk, VA, May 20–24, 2002.

[ref45] Pierce T.; et al. Influence of Increased Isoprene Emissions on Regional Ozone Modeling. J. Geophys. Res.: Atmos. 1998, 103 (D19), 25611–25629. 10.1029/98JD01804.

[ref46] Kim S.-W.; McDonald B. C.; Baidar S.; Brown S. S.; Dube B.; Ferrare R. A.; Frost G. J.; Harley R. A.; Holloway J. S.; Lee H.-J.; McKeen S. A.; Neuman J. A.; Nowak J. B.; Oetjen H.; Ortega I.; Pollack I. B.; Roberts J. M.; Ryerson T. B.; Scarino A. J.; Senff C. J.; Thalman R.; Trainer M.; Volkamer R.; Wagner N.; Washenfelder R. A.; Waxman E.; Young C. J. Modeling the Weekly Cycle of NO_x_ and CO Emissions and Their Impacts on O_3_ in the Los Angeles-South Coast Air Basin during the CalNex 2010 Field Campaign. J. Geophys. Res.: Atmos. 2016, 121 (3), 1340–1360. 10.1002/2015JD024292.

[ref47] Scott K. I.; Benjamin M. T. Development of a Biogenic Volatile Organic Compounds Emission Inventory for the SCOS97-NARSTO Domain. Atmos. Environ. 2003, 37, 39–49. 10.1016/S1352-2310(03)00381-9.

[ref48] Nussbaumer C. M.; Place B. K.; Zhu Q.; Pfannerstill E. Y.; Wooldridge P.; Schulze B. C.; Arata C.; Ward R.; Bucholtz A.; Seinfeld J. H.; Goldstein A. H.; Cohen R. C. Measurement Report: Airborne Measurements of NO_x_ Fluxes over Los Angeles during the RECAP-CA 2021 Campaign, Preprint; Gases/Field Measurements/Troposphere/Chemistry (chemical composition and reactions), 2023 10.5194/egusphere-2023-601.

[ref49] Zhu Q.; Place B.; Pfannerstill E. Y.; Tong S.; Zhang H.; Wang J.; Nussbaumer C. M.; Wooldridge P.; Schulze B. C.; Arata C.; Bucholtz A.; Seinfeld J. H.; Goldstein A. H.; Cohen R. C. Direct Observations of NO_x_ Emissions over the San Joaquin Valley Using Airborne Flux Measurements during RECAP-CA 2021 Field Campaign, Preprint; Gases/Field Measurements/Troposphere/Chemistry (chemical composition and reactions), 2023 10.5194/acp-2023-3.

[ref50] Thornton J. A.; Wooldridge P. J.; Cohen R. C. Atmospheric NO2: In Situ Laser-Induced Fluorescence Detection at Parts per Trillion Mixing Ratios. Anal. Chem. 2000, 72 (3), 528–539. 10.1021/ac9908905. 10695138

[ref51] Day D. A.; Wooldridge P. J.; Dillon M. B.; Thornton J. A.; Cohen R. C. A Thermal Dissociation Laser-Induced Fluorescence Instrument for in Situ Detection of NO2, Peroxy Nitrates, Alkyl Nitrates, and HNO3. J. Geophys. Res.: Atmos. 2002, 107 (D6), ACH 4–1–ACH 4–14. 10.1029/2001JD000779.

[ref52] Dunlea E. J.; Herndon S. C.; Nelson D. D.; Volkamer R. M.; San Martini F.; Sheehy P. M.; Zahniser M. S.; Shorter J. H.; Wormhoudt J. C.; Lamb B. K.; Allwine E. J.; Gaffney J. S.; Marley N. A.; Grutter M.; Marquez C.; Blanco S.; Cardenas B.; Retama A.; Ramos Villegas C. R.; Kolb C. E.; Molina L. T.; Molina M. J. Evaluation of Nitrogen Dioxide Chemiluminescence Monitors in a Polluted Urban Environment. Atmos. Chem. Phys. 2007, 7 (10), 2691–2704. 10.5194/acp-7-2691-2007.

[ref53] Fehsenfel F. C.; Dickerson R. R.; Hübler G.; Luke W. T.; Nunnermacker L. J.; Williams E. J.; Roberts J. M.; Calvert J. G.; Curran C. M.; Delany A. C.; Eubank C. S.; Fahey D. W.; Fried A.; Gandrud B. W.; Langford A. O.; Murphy P. C.; Norton R. B.; Pickering K. E.; Ridley B. A. A Ground-Based Intercomparison of NO, NO_x_, and NO_y_ Measurement Techniques. J. Geophys. Res.: Atmos. 1987, 92 (D12), 14710–14722. 10.1029/Jd092id12p14710.

[ref54] Lambert J.-C.; Keppens A.; Compernolle S.; Eichmann K.-U.; de Graaf M.; Hubert D.; Kleipool Q.; Langerock B.; Sha M. K.; Verhoelst T.; Wagner T.; Ahn C.; Argyrouli A.; Balis D.; Chan K. L.; De Smedt I.; Eskes H.; Fjæraa A. M.; Garane K.; Gleason J. F.; Goutaiil F.; Granville J.; Hedelt P.; Heue K.-P.; Jaross G.; Koukouli M. L.; Landgraf J.; Lutz R.; Nanda S.; Neimeijer S.; Pazmiño A.; Pinardi G.; Pommereau J.-P.; Richter A.; Rozemeijer N.; Sneep M.; Stein Zweers D.; Theys N.; Tilstra G.; Torres O.; Valks P.; van Geffen J.; Vigouroux C.; Wang P.; Weber M. Quarterly Validation Report of the Copernicus Sentinel-5 Precursor Operational Data Products #15, S5P MPC Routine Operations Consolidated Validation Report series, Issue #15, April 2018–May 2022, 2021.

[ref55] Li M.; McDonald B. C.; McKeen S. A.; Eskes H.; Levelt P.; Francoeur C.; Harkins C.; He J.; Barth M.; Henze D. K.; Bela M. M.; Trainer M.; de Gouw J. A.; Frost G. J. Assessment of Updated Fuel-Based Emissions Inventories Over the Contiguous United States Using TROPOMI NO2 Retrievals. J. Geophys. Res.: Atmos. 2021, 126 (24), e2021JD035484 10.1029/2021JD035484.

[ref56] Eskes H.; Geffen J. van.; Boersma F.; Eichmann K.-U.; Apituley A.; Pedergnana M.; Sneep M.; Veefkind J. P.; Loyola D.; Royal Netherlands Meteorological Institute. Sentinel-5 Precursor/TROPOMI Level 2 Product User Manual Nitrogendioxide. 2022.

[ref57] Lamsal L. N.; Martin R. V.; van Donkelaar A.; Celarier E. A.; Bucsela E. J.; Boersma K. F.; Dirksen R.; Luo C.; Wang Y. Indirect Validation of Tropospheric Nitrogen Dioxide Retrieved from the OMI Satellite Instrument: Insight into the Seasonal Variation of Nitrogen Oxides at Northern Midlatitudes. J. Geophys. Res.: Atmos. 2010, 115 (D5), D05302 10.1029/2009JD013351.

[ref58] Judd L. M.; Al-Saadi J. A.; Szykman J. J.; Valin L. C.; Janz S. J.; Kowalewski M. G.; Eskes H. J.; Veefkind J. P.; Cede A.; Mueller M.; Gebetsberger M.; Swap R.; Pierce R. B.; Nowlan C. R.; Abad G. G.; Nehrir A.; Williams D. Evaluating Sentinel-5P TROPOMI Tropospheric NO_2_ Column Densities with Airborne and Pandora Spectrometers near New York City and Long Island Sound. Atmos. Meas. Technol. 2020, 13 (11), 6113–6140. 10.5194/amt-13-6113-2020. PMC8193800 34122664

[ref59] California Air Resources Board. EMFAC Emissions Database. https://arb.ca.gov/emfac/ (accessed May 31, 2023).

[ref60] Bluffstone R. A.; Ouderkirk B. Warehouses, Trucks, and Pm2.5: Human Health and Logistics Industry Growth in the Eastern Inland Empire. Contemp. Econ. Policy 2007, 25 (1), 79–91. 10.1111/j.1465-7287.2006.00017.x.

[ref61] Redford Conservancy at Pitzer College. Warehouse CITY. https://radicalresearch.shinyapps.io/WarehouseCITY/ (accessed June 15, 2023).

