# Supplementary material for: Improved Spatial Resolution in Modeling of Nitrogen Oxide Concentrations in the Los Angeles Basin
Source: Environ Sci Technol. 2023 Nov 30;57(49):20689–98. doi: 10.1021/acs.est.3c06158 (PMC10720381; doi:10.1021/acs.est.3c06158)
Supplement: Supplementary file 1 — es3c06158_si_001.pdf [file es3c06158_si_001.pdf]

Improved Spatial Resolution in Modeling of Nitrogen Oxide Concentrations in the Los Angeles Basin

Katelyn A. Yu[1,2], Meng Li[2,3], Colin Harkins[2,3], Jian He[2,3], Qindan Zhu[2,4,†], Bert Verreyken[2,††], Rebecca H. Schwantes[2], Ronald C. Cohen[4], Brian C. McDonald[2], Robert A. Harley[1,*]

1. Department of Civil and Environmental Engineering, University of California, Berkeley, Berkeley, CA, 94720, USA
2. Chemical Sciences Laboratory, NOAA Earth System Research Laboratories, Boulder, CO, 80305, USA
3. Cooperative Institute for Research in Environmental Sciences, University of Colorado, Boulder, CO, 80309, USA
4. Department of Chemistry, University of California, Berkeley, Berkeley, CA, 94720, USA

*Corresponding Author: Robert A. Harley (harley@ce.berkeley.edu)

Summary of Supporting Information:

Number of pages: 11

Number of figures: 7

Number of tables: 1

Table S1: Summary of WRF-Chem model setup and input data

WRF-Chem model version	WRFv4.2.2
Horizontal resolution	D01: 4km x 4km over California and Nevada<br>D02: 1.3km x 1.3km over southern California
Vertical resolution	50 vertical levels from surface to 50 hPa
Initial/boundary conditions (meteorology)	High Resolution Rapid Refresh (HRRR) at 3 km resolution[1]
Initial/Boundary conditions (chemistry)	12 km resolution WRF-Chem run using NAM meteorology and chemical boundary conditions from the Realtime Air Quality Modeling System (RAQMS)
Gas phase chemistry	RACM-ESRL-VCP[2]
Photolysis scheme	Madronich photolysis (TUV)
Anthropogenic emissions	Modified Fuel-based Inventory of Vehicle Emissions (FIVE)[3], Fuel-based oil and gas (FOG) inventory[4], Continuous Emission Monitoring Systems (CEMS)[5], NEI 17 scaled to 2021[6], Copernicus Atmosphere Monitoring Service (CAMS)[7]
Biogenic emissions	Biogenic Emissions Inventory System (BEIS3.14)[8] with updated isoprene and monoterpenes from the urban land cover type[9,10]
Additional parameterizations	Mellor-Yamada Nakanishi and Niino surface layer, Mellor-Yamada Nakanishi and Niino level 2.5 planetary boundary layer, Grell-Devenyi (GD) ensemble cumulus, Noah land surface model

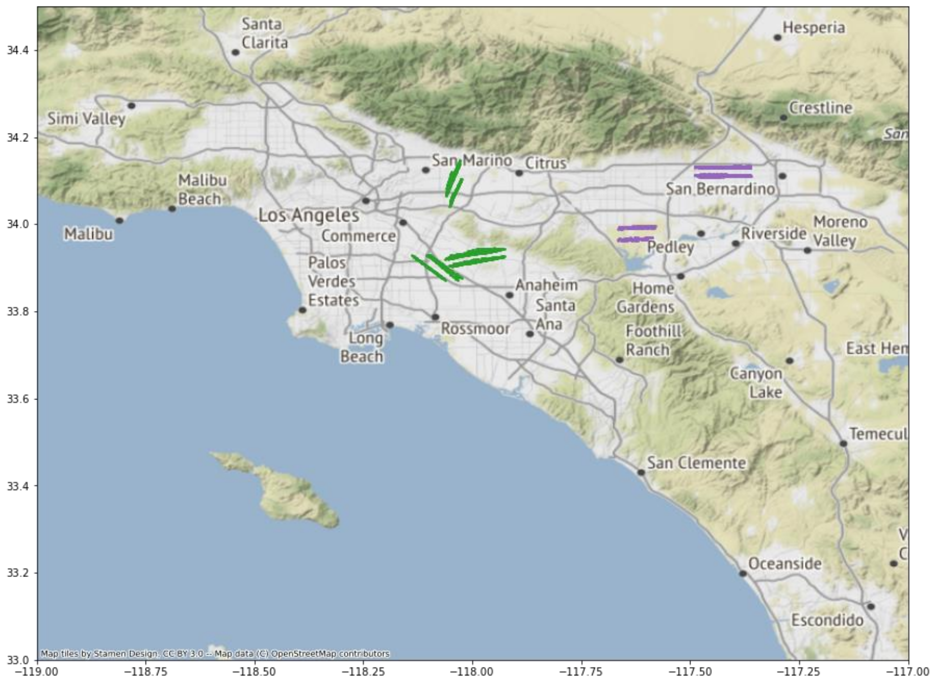

Figure S1: Flight paths used for vertical profile comparison. NO_2 measured in LA West (green) are separated from measurements taken in LA East (purple) to account for spatial differences in temperature.

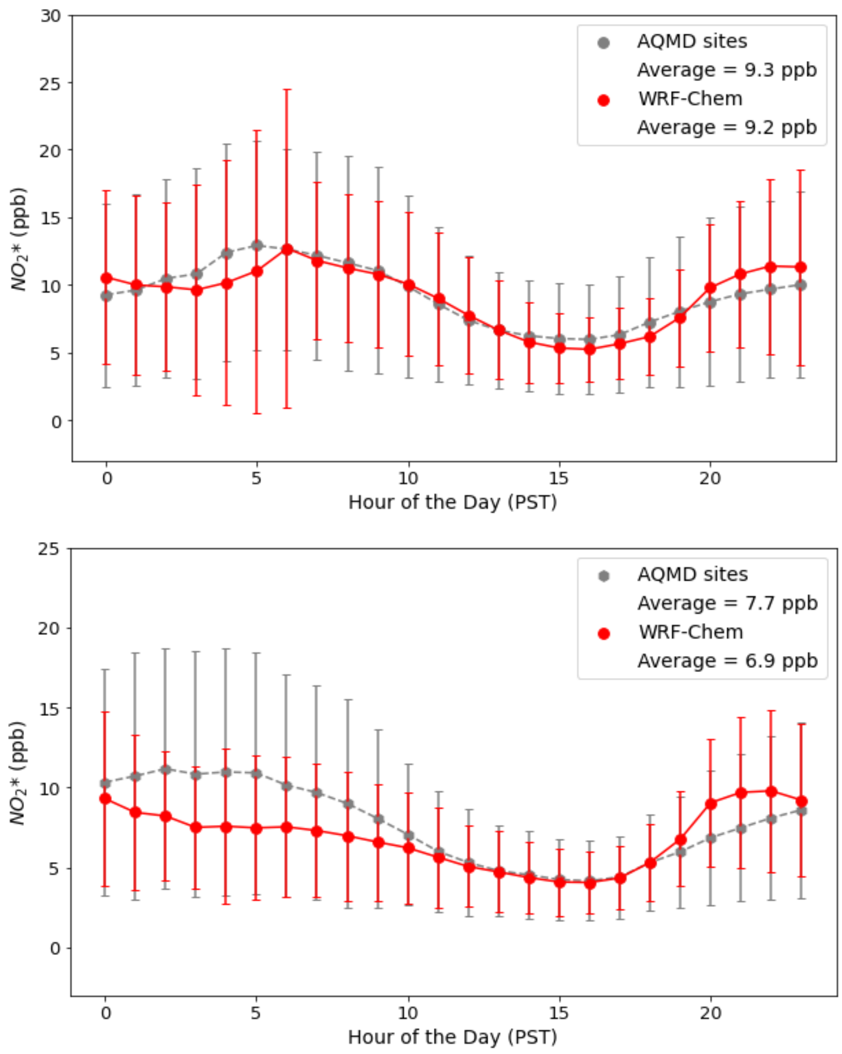

Figure S2: Diurnal variation of NO_2* (NO_2* = NO_2 + PAN + alkyl nitrates + HONO + 2*N_2O_5) from WRF-Chem compared to NO_2 from 24 AQMD surface monitors in the South Coast Air Basin on weekdays (top) and weekends (bottom) for June 2021. Error bars show one standard deviation.

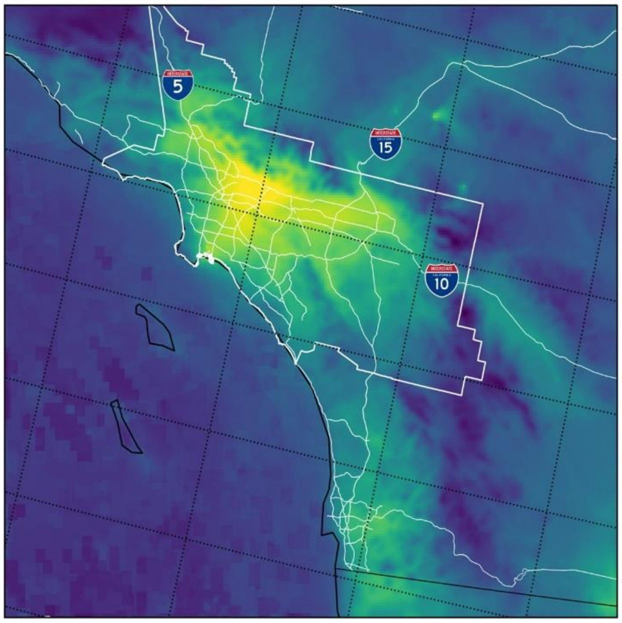

Figure S3: Key interstate freeways within the Los Angeles domain for spatial reference

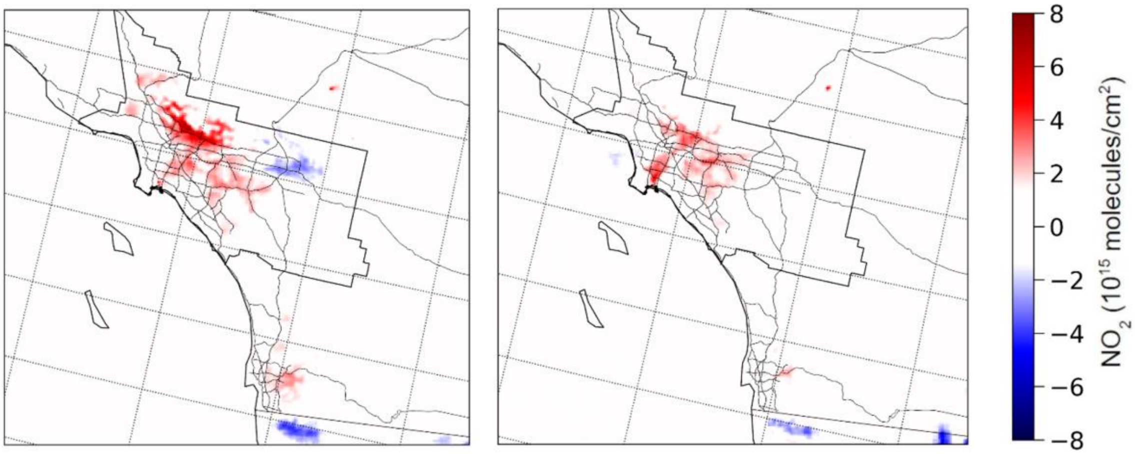

Figure S4: Average WRF-Chem vertical NO_2 columns minus average TROPOMI vertical NO_2 columns on weekdays (left) and weekends (right) for June 2021.

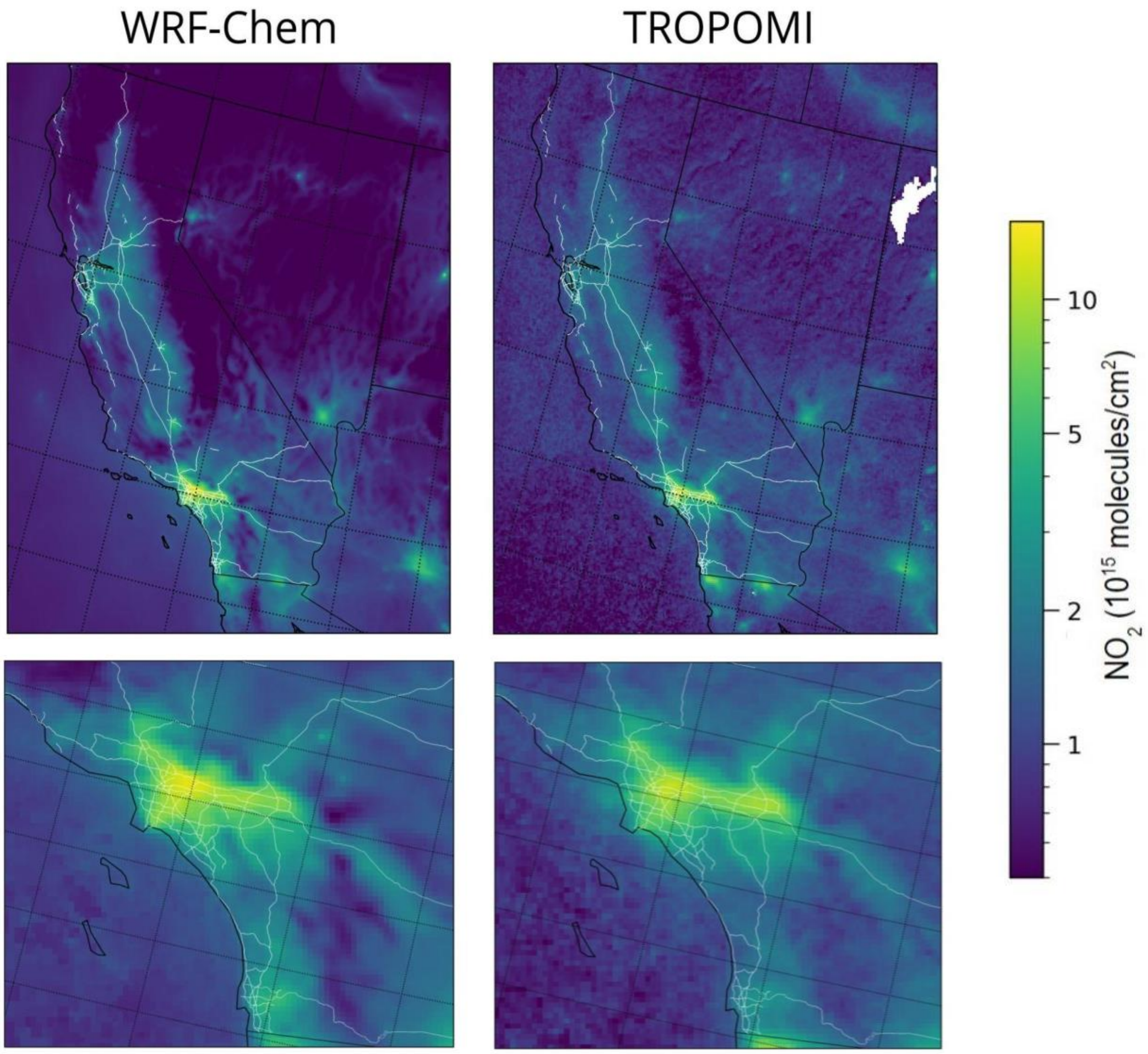

Figure S5: Average tropospheric NO_2 columns at 4 km resolution over California for June 2021, predicted using the WRF-Chem model with comparisons to TROPOMI satellite data. The top panels show the entire CA/NV domain, while the bottom panels show the zoomed in Los Angeles domain. Major highways are shown in white.

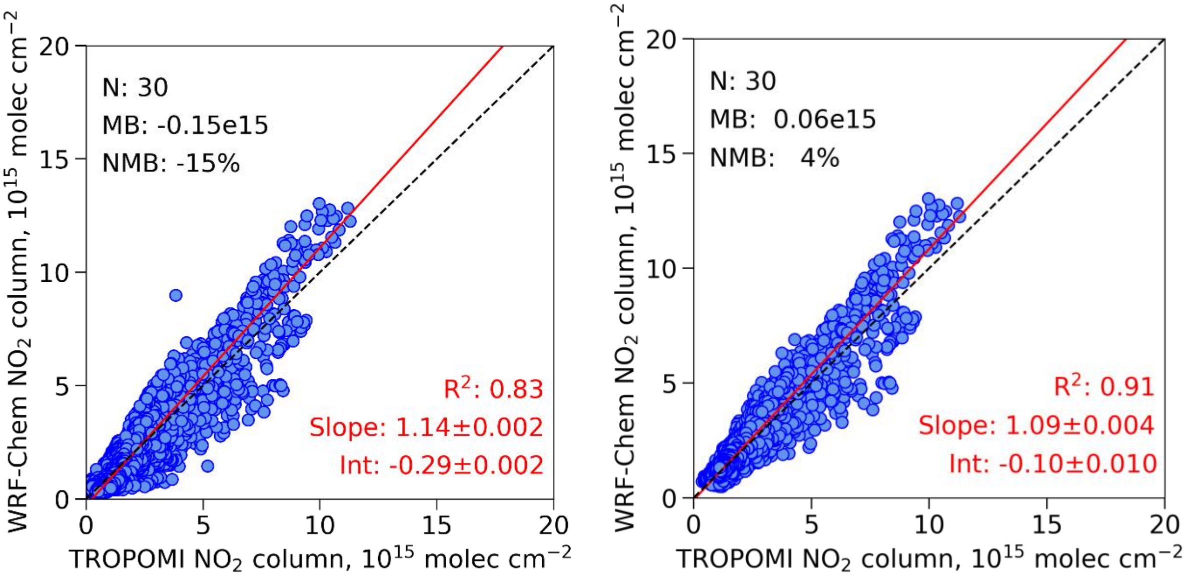

Figure S6: Orthogonal distance regression between modeled and satellite-derived NO_2 columns at 4 km resolution the full California domain (left panel) and the southern California domain (right panel) in June 2021.

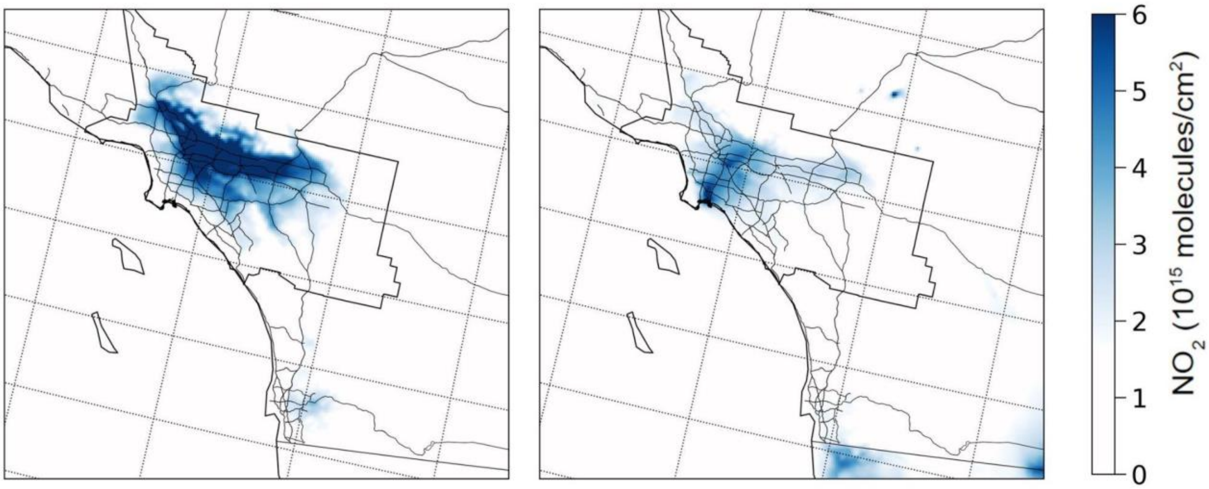

Figure S7: Weekday-weekend tropospheric NO_2 column difference of on-road sector (left) and the remaining combined offroad, area, and point sectors (right)

Present address: †Qindan Zhu: Department of Earth, Atmospheric and Planetary Sciences, Massachusetts Institute of Technology, Cambridge, MA, 02139, USA

Present address: ††Bert Verreyken: Royal Belgian Institute for Space Aeronomy (BIRA-IASB), Ukkel, Belgium and Gembloux Agro-Biotech, University of Liège, Gembloux, 4000, Belgium

References

(1) Dowell, D. C.; Alexander, C. R.; James, E. P.; Weygandt, S. S.; Benjamin, S. G.; Manikin, G. S.; Blake, B. T.; Brown, J. M.; Olson, J. B.; Hu, M.; Smirnova, T. G.; Ladwig, T.; Kenyon, J. S.; Ahmadov, R.; Turner, D. D.; Duda, J. D.; Alcott, T. I. The High-Resolution Rapid Refresh (HRRR): An Hourly Updating Convection-Allowing Forecast Model. Part I: Motivation and System Description. *Weather Forecast.* **2022**, *37* (8), 1371–1395. https://doi.org/10.1175/WAF-D-21-0151.1.

(2) Coggon, M. M.; Gkatzelis, G. I.; McDonald, B. C.; Gilman, J. B.; Schwantes, R. H.; Abuhassan, N.; Aikin, K. C.; Arend, M. F.; Berkoff, T. A.; Brown, S. S.; Campos, T. L.; Dickerson, R. R.; Gronoff, G.; Hurley, J. F.; Isaacman-VanWertz, G.; Koss, A. R.; Li, M.; McKeen, S. A.; Moshary, F.; Peischl, J.; Pospisilova, V.; Ren, X.; Wilson, A.; Wu, Y.; Trainer, M.; Warneke, C. Volatile Chemical Product Emissions Enhance Ozone and Modulate Urban Chemistry. *Proc. Natl. Acad. Sci.* **2021**, *118* (32), e2026653118. https://doi.org/10.1073/pnas.2026653118.

(3) Harkins, C.; McDonald, B. C.; Henze, D. K.; Wiedinmyer, C. A Fuel-Based Method for Updating Mobile Source Emissions during the COVID-19 Pandemic. *Environ. Res. Lett.* **2021**, *16* (6), 065018. https://doi.org/10.1088/1748-9326/ac0660.

(4) Francoeur, C. B.; McDonald, B. C.; Gilman, J. B.; Zarzana, K. J.; Dix, B.; Brown, S. S.; de Gouw, J. A.; Frost, G. J.; Li, M.; McKeen, S. A.; Peischl, J.; Pollack, I. B.; Ryerson, T. B.; Thompson, C.; Warneke, C.; Trainer, M. Quantifying Methane and Ozone Precursor Emissions from Oil and Gas Production Regions across the Contiguous US. *Environ. Sci. Technol.* **2021**, *55* (13), 9129–9139. https://doi.org/10.1021/acs.est.0c07352.

(5) U.S. Environmental Protection Agency. *Continuous Emission Monitoring Systems*. https://www.epa.gov/emc/emc-continuous-emission-monitoring-systems (accessed 2023-05-31).

(6) U.S. Environmental Protection Agency. National Emissions Inventory (NEI) 2017, April 2020 Version. https://gispub.epa.gov/neireport/2017/ (accessed 2023-05-31).

(7) Granier, C.; Darras, S.; Denier van der Gon, H.; Doubalova, J.; Elguindi, N.; Galle, B.; Gauss, M.; Guevara, M.; Jalkanen, J.-P.; Kuenen, J.; Liousse, C.; Quack, B.; Simpson, D.; Sindelarova, K. The Copernicus Atmosphere Monitoring Service Global and Regional Emissions (April 2019 Version). https://doi.org/10.24380/D0BN-KX16.

(8) Pierce, T.; Geron, C.; Pouliot, G.; Kinnee, E.; Vukovich, J. Integration of the Biogenic Emissions Inventory System (BEIS3) into the Community Multiscale Air Quality Modeling System. Norfolk, VA.

(9) Scott, K. I.; Benjamin, M. T. Development of a Biogenic Volatile Organic Compounds Emission Inventory for the SCOS97-NARSTO Domain. *Atmos. Environ.* **2003**, *37*, 39–49. https://doi.org/10.1016/S1352-2310(03)00381-9.

(10) Kim, S.-W.; McDonald, B. C.; Baidar, S.; Brown, S. S.; Dube, B.; Ferrare, R. A.; Frost, G. J.; Harley, R. A.; Holloway, J. S.; Lee, H.-J.; McKeen, S. A.; Neuman, J. A.; Nowak, J. B.; Oetjen, H.; Ortega, I.; Pollack, I. B.; Roberts, J. M.; Ryerson, T. B.; Scarino, A. J.; Senff, C. J.; Thalman, R.; Trainer, M.; Volkamer, R.; Wagner, N.; Washenfelder, R. A.; Waxman, E.; Young,

C. J. Modeling the Weekly Cycle of NO_x and CO Emissions and Their Impacts on O_3 in the Los Angeles-South Coast Air Basin during the CalNex 2010 Field Campaign. *J. Geophys. Res. Atmospheres* **2016**, *121* (3), 1340–1360. https://doi.org/10.1002/2015JD024292.
